# A Hands‐On Approach to Learning: Gesture Production During Encoding and its Effect on Narrative Recall

**DOI:** 10.1111/cogs.13214

**Published:** 2022-12-08

**Authors:** Avni Bharadwaj, Nicole Dargue, Naomi Sweller

**Affiliations:** ^1^ School of Psychological Sciences Macquarie University; ^2^ Autism Centre of Excellence, School of Education and Professional Studies Griffith University

**Keywords:** Narrative recall, Representational gesture, Deictic gesture, Beat gesture, Learning, Communication, Individual differences, Memory

## Abstract

Research has shown that gesture production supports learning across a number of tasks. It is unclear, however, whether gesture production during encoding can support narrative recall, who gesture production benefits most, and whether certain types of gestures are more beneficial than others. This study, therefore, investigated the effect of gesture production during the encoding of a narrative on subsequent narrative recall, and whether individuals’ levels of verbal and nonverbal memory moderated this effect. Additionally, this study investigated whether producing certain types of gestures during encoding was more beneficial than others. Participants (*N* = 90, *M_age_* = 20.43) read aloud a narrative while under instruction to produce gestures, under no specific instruction to produce gestures, or were required to keep their hands behind their back to prevent them from gesturing. Participants completed measures assessing verbal and nonverbal memory. While gesture production during encoding benefitted narrative recall (as measured through specific questions), verbal memory moderated the effect, such that gesture production was more beneficial for individuals with higher than lower verbal memory. Furthermore, producing representational gestures during encoding benefitted recall of points in the narrative at which those gestures were produced, while beat gestures had no effect. Findings have implications for understanding the mechanisms underlying the links between gesture and learning, as well as practical implications in instructional settings.

## 1 Introduction

Gestures are an integral component of interpersonal communication and provide nonverbal information in addition to, or in place of, verbal communication (McNeill, 1992). Gestures are ubiquitous and widely produced, including by people who are blind from birth, and even when there is no audience present (Alibali, Heath, & Myers, 2001). The widespread production of gestures even in the absence of any possible effect on an observer, suggests that gesture production is likely to be beneficial to the speaker producing the gestures themselves. Indeed, evidence suggests producing gestures assists in learning languages (Macedonia & Knösche, 2011; Sweller, Shinooka‐Phelan, & Austin, 2020), problem‐solving (Alibali, Spencer, Knox, & Kita, 2011; Chu & Kita, 2011; Goldin‐Meadow, Nusbaum, Kelly, & Wagner, 2001; Kita, Alibali, & Chu, 2017), speech production and communication (Driskell & Radtke, 2003; McNeil, Alibali, & Evans, 2000; Rauscher et al., 1996), and narrative comprehension and recall (Alibali et al., 2001; Dargue & Sweller, 2020; Dargue, Sweller, & Jones, 2019). While research has been conducted on gesture observation and narrative recall (e.g., Aussems & Kita, 2019; Dargue & Sweller, 2018a, 2018b, 2020; Llanes‐Coromina, Vilà‐Giménez, Kushch, Borràs‐Comes, & Prieto, 2018), the current study focuses on the effect of gesture production. Narrative recall refers to individuals’ ability to understand and recall narratives and stories (Bóna, 2013). This ability is key for social interactions and learning (Bóna, 2013). As such, understanding whether gesture production may enhance narrative recall could have important implications for learning.

Although a wealth of prior research has examined the effects of gesture production on mathematics‐based tasks (Cook, 2018; Cook, Yip, & Goldin‐Meadow, 2012; Goldin‐Meadow et al., 2001; Wagner, Mitchell, & Goldin‐meadow, 2008), studies examining the effects of gesture production on narrative recall are sparse, with inconsistent findings (Cameron & Xu, 2011; Dargue & Sweller, 2020; Dargue et al., 2019; Vilà‐Giménez & Prieto, 2020). While Cameron and Xu (2011) found that gesture production was beneficial for recall, Dargue and Sweller (2020) found that gesture production had no benefit. Such inconsistent findings raise the question of whether factors such as individual differences in cognitive abilities and the type of gesture produced moderate effects of gesture production on narrative recall. Understanding whether gesture production benefits narrative recall and under what circumstances has important implications for our knowledge of learning processes. There are also important practical applications, including the implementation of gesture‐based learning strategies. The current study therefore examines the effects of gesture production on narrative recall, and whether such effects are moderated by individual differences in memory (verbal and nonverbal) and the type of gesture produced.

### 1.1 Gesture types

Gestures can be classified in four different ways: iconic, metaphoric, deictic, and beat (McNeill, 1992). Iconic gestures are semantically related to the content of speech; they depict concrete objects or actions in accompanying speech (McNeill, 1992). A speaker with their hand in a fist with two fingers outstretched moving back and forth to show walking provides an example. Where a gesture depicts an abstract idea or concept, this is known as a metaphoric gesture (McNeill, 1992). For example, a speaker with both palms facing upward and hands moving up and down alternately to indicate weighing up options (as they are not physically weighing options). Iconic and metaphoric gestures are frequently termed representational gestures, as they represent accompanying speech content either concretely (iconic gestures) or abstractly (metaphoric gestures; Kita et al., 2017). Where a gesture indicates the location or direction of an object or event, it is known as a deictic or pointing gesture (McNeill, 1992), such as a speaker pointing up while stating, “look at the clouds”. Unlike iconic, metaphoric, and deictic gestures, beat gestures are limited in their semantic relationship to speech. Such gestures are rhythmic movements of the hands, which may add emphasis to a spoken message (McNeill, 1992), for example, a teacher rhythmically flicking their wrist while explaining a concept. Production of all gesture types has been shown to be beneficial in learning and communication (Cameron & Xu, 2011; Cook et al., 2012; Vilà‐Giménez & Prieto, 2020).

### 1.2 Gesture production and learning

As noted above, gestures are produced universally, even without a present listener (Alibali et al., 2001). Gesture production begins during the infant years, with infants using pointing gestures from 9 to 12 months of age (Cameron & Xu, 2011). The early use of gestures, particularly prior to the development of verbal speech, suggests that gestures have significant benefits for the speaker themselves. Indeed, gestures can assist in learning and carrying out both nonverbal and verbal tasks. The production of speech and gesture together, or the production of only gestures has been effective in supporting the retention of mathematical problem‐solving strategies, compared to only verbal explanations (Wagner et al., 2008). The production of gestures coordinated with speech when explaining math problems has been found to be more beneficial in assisting recall, than hand movements semantically unrelated to accompanying speech (Cook et al., 2012). Gestures also influence strategy choices in spatial problem‐solving, with participants who produced gestures more likely to generate perceptual‐motor strategies than participants who did not produce gestures (Alibali et al., 2011).

With respect to verbal tasks, children produce gestures from a young age, and there is an association between gestures produced between the ages of 10–24 months, and later vocabulary development (Iverson & Goldin‐Meadow, 2005). In a study conducted by Kriegstein, Mayer, Yildiz, and Macedonia (2015), words learned with accompanying gestures were better remembered than when adult participants were shown pictures. Furthermore, gesture production during the encoding process along with audio‐visual methods of encoding, can improve learning. Participants who used gestures during the encoding of word items in a foreign language used more of these items when creating new sentences (Macedonia & Knösche, 2011). Conversely, however, spontaneous gesture production during recall was associated with lower performance on cued recall of Japanese to English verbs (Sweller et al., 2020), indicating mixed results on the impact of gesture production on foreign language learning.

Beyond word learning and foreign language learning, gesture production may benefit narrative recall in certain circumstances. Previous studies by Cameron and Xu (2011), Dargue and Sweller (2020), and Vilà‐Giménez and Prieto (2020) assess the impact of gesture production during the recall of a narrative, or in storytelling, with mixed results on recall performance. Four‐ to five‐year‐old children who produced representational gestures during narrative recall performed better on narrative comprehension than those who did not produce gestures (Cameron & Xu, 2011). Cameron and Xu (2011) also found a beneficial effect of gesture on narrative recall where children simply pointed on a map (i.e., performed a deictic gesture). However, it is difficult to disentangle the effects of gesture observation and production in this study, as participants also observed gestures during encoding. Vilà‐Giménez and Prieto (2020) found that when children between 5 and 6 years of age were encouraged to produce beat gestures when retelling a narrative during a training phase, they performed better at a post‐test when telling a narrative from an image than they had prior to the training phase. This finding indicated that gestures do not need to be semantically linked to speech to be beneficial for learning. Indeed, beat gestures may benefit learning by helping the speaker focus on important aspects of the narrative (Vilà‐Giménez & Prieto, 2021). Contrary to these findings, however, Dargue and Sweller (2020) found no beneficial effects of any gesture production during narrative recall.

The studies noted above by Cameron and Xu (2011) and Dargue and Sweller (2020), however, assessed the impact of gesture production during *recall*, rather than encoding. Wakefield and James (2015) found that gesture production during training when learning palindromes better facilitated learning than speech alone. Furthermore, a meta‐analysis by Dargue et al. (2019) found that studies assessing gesture production at encoding *or* recall showed a greater benefit on comprehension than studies assessing the effect of gesture observation. However, as this meta‐analysis did not differentiate between studies assessing gesture production at encoding or recall, it is unclear whether gesture production at encoding may contribute to this effect to a greater extent than gesture production at recall.

Furthermore, it is unclear whether gesture production supports the recall of a narrative as a whole, or only recall of the specific points of the narrative gestures accompany. Dargue and Sweller (2020) found that gestures only supported the recall of specific points they accompanied and did not support the recall of points that did not have accompanying gestures. It is, however, important to note that the study assessed the benefits of gesture observation, and it is therefore necessary to determine whether gesture production will produce the same effect. The theories underlying why producing gestures may benefit learning (described below) center on the effects of gesture production during the learning process (i.e., encoding) itself, rather than during retrieval. It is therefore possible that the mixed results seen in previous studies stem at least in part from the gesture production not occurring when it may be best placed to aid learning. That is, that gesture production did not occur during encoding.

### 1.3 Underlying mechanisms

There are several potential mechanisms through which gesture production may assist in learning and recall (see Cook, 2018 for a review). Gestures may benefit learning through perceptual, attentional, linguistic, spatial, memory, and embodied mechanisms (Cook, 2018). Although these proposed mechanisms are by no means mutually exclusive (i.e., more than one mechanism may be operating at any time), the final two, memory and embodied mechanisms, are central to the current study.

A key theory is that gesture production allows for cognitive resources to be divided in a manner that decreases the load on working memory, leading to improved task performance (Goldin‐Meadow et al., 2001). Gestures often convey additional information to the content of speech (Goldin‐Meadow & Alibali, 2014), and do so in a visuospatial format, allowing information to be better encoded through two modalities (Goldin‐Meadow et al., 2001; Özer & Göksun, 2020a). This may reduce the cognitive burden of processing information, thereby alleviating cognitive load. Indeed, speakers gesture more when communicating information not present in the immediate environment, suggesting that the gestures may help to maintain that information in working memory (Cook, 2018). The beneficial effect of gestures on working memory can also be compounded through the use of gestures that are semantically related to speech content (Cook et al., 2012). Cook et al. (2012) found that participants who produced meaningful gestures when explaining solutions to a math problem performed better on a concurrent memory task than those who produced meaningless gestures or no gestures. This finding provides support for the notion that gesture lightens the working memory load, as meaningful gestures provide information in a different modality than speech, freeing resources in working memory (Cook et al., 2012).

Another potential mechanism through which gesture production may be facilitated and assist in learning is through the activation of motor systems when people think about (or see) motor actions, including gestures (Hostetter & Alibali, 2019). According to the Gesture as Simulated Action (GSA) framework, gestures embody and represent motor activity that occurs when people think and speak (Hostetter & Alibali, 2019). The framework suggests that as a person speaks, areas of the brain responsible for the activity being spoken about are activated, and the speaker will gesture when those areas are activated past the speaker's individual gesture threshold. The gesture threshold is the level of resistance of a person to producing gestures and is influenced by a number of factors. For example, a person's gesture threshold may be high if they believe that gestures are impolite, meaning they may be less likely to gesture. In contrast, an individual's gesture threshold might be low if they believe gestures are helpful for communication, meaning they may be more likely to gesture. Individuals with higher gesture thresholds are less likely to gesture than those with low thresholds.

Mental simulations occur when speakers express their thoughts, particularly when those thoughts are visual in nature (e.g., when thinking of a person watering their garden; Hostetter & Alibali, 2019). Where speakers rely on stored verbal memory, however, such as when recalling a list of words, they are less likely to produce mental simulations and thus also less likely to produce gestures (Hostetter & Alibali, 2019). In addition to gesture production being more likely when thinking about actions or visual images, gesture production may also elicit or enhance visual representations (Beilock & Goldin‐Meadow, 2010). In a study by Beilock and Goldin‐Meadow (2010), participants completed the Tower of Hanoi task, explained their reasoning, and completed the task again while following actions that were either consistent or inconsistent with gestures produced when explaining the task. When actions were inconsistent with gestures produced when explaining the task, participants performed more poorly on the task than when the actions were consistent with gestures produced. Therefore, gesture production may be more likely to occur when mental simulations are activated, but may also further enhance mental representations of the speaker and support learning. Although the present study was not designed to directly differentiate between the idea that gestures reduce cognitive load and the GSA framework to determine which mechanisms may underlie learning when gesture production occurs during encoding, the effects of gesture production at encoding on narrative recall have the potential to provide support for these theories.

The literature reviewed above has only explored the effects of gesture in general. It is possible, however, that gestures are not equally beneficial for everyone. If, for example, producing gesture alleviates the load on working memory, perhaps gestures will be differentially beneficial for individuals with varying levels of working memory capacity. There is little research to date which examines individual differences, such as working memory capacity, in relation to the effects of gesture on learning. Furthermore, the limited research that has been conducted in this area has focused on the moderating effect of individual differences on the benefits of observing, rather than producing, gestures (McKern, Dargue, Sweller, Kazuki, & Austin, 2021).

### 1.4 Gesture and verbal memory

Gestures may support verbal working memory, potentially by decreasing cognitive load. It has been proposed that speakers are likely to perform better at word list recall when they gesture during description tasks than when they do not gesture, as gestures allow cognitive resources to be distributed between spoken and visual modalities (Cook et al., 2012). Furthermore, as gestures have been proposed to lighten the cognitive load associated with a task, it has been suggested that those with lower levels of verbal memory are likely to produce more gestures (Gillespie, James, Federmeier, & Watson, 2014). Wagner, Nusbaum, and Goldin‐meadow (2004) had participants explain math tasks while concurrently holding a string of letters in memory. Participants remembered more letters when they were allowed to gesture while explaining the math problems, as compared with when they were prevented from gesturing. Similarly, Alibali, Kita, Young, Alibali, and Young (2010) found that during spatial problem‐solving explanations, participants produced more gestures during explanation than description tasks, and suggested that this is especially the case where verbalization is difficult. On the other hand, Cravotta, Prieto, and Busa (2021) found that restricting gesture production when retelling a narrative did not affect verbal speech. Cravotta et al. (2021), therefore, suggest that restricting gesture production may have differential effects on individuals, depending on their need for gestures when speaking. If gestures indeed lighten the load on verbal working memory by engaging other modalities, performance on verbal memory tasks is likely to improve more for individuals who have lower verbal memory capacity than those with higher verbal memory capacity (Goldin‐Meadow et al., 2001).

### 1.5 Gesture and nonverbal memory

In addition to supporting verbal memory performance, gestures may also improve performance on nonverbal memory tasks. In the context of the current study, nonverbal memory involves recalling and recreating visual or spatial events or images (Luzzi et al., 2011). According to the GSA framework, people are likely to produce gestures when they engage their motor systems (Hilverman, Cook, & Duff, 2018). This activation of motor systems may facilitate vivid nonverbal mental representations (Alibali et al., 2011). That is, the act of producing gestures may result in more vivid nonverbal mental representations (such as visual or proprioceptive) than when gestures are not produced, reducing cognitive load by dividing resources across verbal and visual modalities (Chu, Meyer, Foulkes, & Kita, 2014; Özer & Göksun, 2020a). In turn, these richer mental representations may facilitate performance on nonverbal working memory tasks (Chu et al., 2014). This suggestion is supported by findings that those with lower levels of nonverbal (visual) memory produced more representational gestures than those with better nonverbal memory capacity (Chu et al., 2014). Furthermore, the motor simulations generated prior to gesture production have been demonstrated to add to an individual's visual representations (Beilock & Goldin‐Meadow, 2010), thereby enhancing learning. As a result, those with poorer levels of nonverbal memory may benefit more from gesture production. If an individual finds a task difficult due to their lower nonverbal memory capacity, there is more potential for gesture production to benefit learning by enhancing and adding to mental representations, than for an individual who perhaps struggles less with the task.

### 1.6 Present study

If gesture indeed helps to alleviate the load on working memory, the benefit of gesture production may be strongest during the learning or encoding phase, rather than the retrieval phase of a narrative learning task. Furthermore, it is also possible that gestures may be differentially helpful for those with different levels of verbal and nonverbal memory. The current study, therefore, aimed to assess the following:

1. Whether gesture production during encoding of a narrative assists later recall; and whether the impact of gesture production is dependent on levels of verbal and nonverbal memory.
2. Given the inconsistent findings of existing research on the impacts of different types of gestures on narrative comprehension or recall (Cameron & Xu, 2011; Dargue & Sweller, 2020; Vilà‐Giménez & Prieto, 2020), this study examined whether there were differential effects of the production of different types of gesture on narrative recall.
3. Finally, although previous findings have shown that gesture observation primarily benefits the recall of specific points in a narrative that are accompanied by gestures (Dargue & Sweller, 2020), it is yet to be determined whether this holds true for gesture production. Thus, the current study investigated whether gesture production at encoding benefited recall of the narrative as a whole, or only the specific points at which the gesture was produced.

Participants read aloud a narrative while producing hand movements (Instructed Gesture condition), holding their hands behind their back to prevent gesturing (No Gesture condition), or while given no instruction regarding hand movements (Spontaneous Gesture condition). Participants’ verbal and nonverbal memory were assessed as separate, continuous measures to determine whether verbal and nonverbal memory ability moderate the effect of gesture production on recall. The recall was assessed through free recall and cued recall of specific points in the narrative.

Given existing research suggests gesture production alleviates the load on verbal working memory by lowering the cognitive load, and nonverbal working memory by adding to mental representations (Beilock & Goldin‐Meadow, 2010; Chu et al., 2014; Cook et al., 2012; Wagner et al., 2004), it was expected that producing gestures during encoding would benefit narrative recall. Those in the Instructed Gesture condition were expected to recall more of the narrative than those in the Spontaneous and No Gesture conditions, and those in the Spontaneous Gesture condition were expected to recall more than those in the No Gesture condition. Furthermore, it was expected that gesture production at encoding would benefit individuals with lower verbal and nonverbal memory more than those with higher verbal and nonverbal memory. Finally, for cued recall specifically (as measured through specific questions), participants were expected to recall more for specific points of the narrative where they produced a gesture at encoding than for points where they did not produce a gesture at encoding. Although this relationship could similarly hold for free recall, it is less clear for free recall than for cued recall exactly to which point in the narrative participants are referring. This is because there are multiple similar events in the narrative, such as the letters flying around. Direct matching of gestures performed during encoding and free recall is, therefore, less reliable than matching for specific questions. This final hypothesis was therefore only addressed in relation to the specific recall questions.

## 2 Method

### 2.1 Participants

A total of 99 undergraduate psychology students were recruited via advertisement on the Macquarie University Psychology Participant Pool. Based on previous meta‐analyses which both showed Cohen's *d* of 0.61 for the effect of gesture (Dargue et al., 2019; Hostetter, 2011), a sample size of 107 was needed for the current analyses. Due to participant unavailability, only 99 participants were able to be recruited. However, nine students were excluded from the final analyses due to experimenter error or technological difficulties. The final sample consisted of 90 students (73 females and 17 males), ranging from 17 to 32 years of age (*M* = 20.43, *SD* = 2.87). As participants self‐recruited from an undergraduate psychology population, there was a large gender imbalance between males and females in the sample. Given this imbalance and the relatively small number of males recruited, it was not possible to check for gender effects. The study had a between‐subjects design, with participants pseudo‐randomly allocated (to ensure equal allocation to conditions) to one of three gesture conditions. There were 30 students in each condition, with 26 females and four males in both the Instructed and Spontaneous Gesture conditions, and 21 females and nine males in the No Gesture condition. Three participants, one from each condition, were excluded from analyses which included the total specific questions score due to partially missing data but were included in all other analyses. Participants provided informed consent prior to participation via Qualtrics.

### 2.2 Materials and procedure

The Rey–Osterrieth Complex Figure Test (RCFT; Meyers & Meyers, 1995) was used to assess participants’ nonverbal memory, and was administered and scored by the experimenter according to standardized instructions. The Rey Auditory Verbal Learning Test (RAVLT; Rey, 1941) was used to assess participants’ verbal memory, and was administered and scored by the experimenter consistent with standardized procedures (see Strauss, Sherman, & Spreen, 2006 for procedure). For both the RCFT and RAVLT, only the results for the immediate and delayed trials were used in the final analysis, as these trials have been demonstrated to measure nonverbal and verbal memory retention respectively (Strauss et al., 2006). Internal reliability for the RCFT is acceptable (above .80 for recall conditions; Berry, Allen, & Schmitt, 1991). Internal reliability for the RAVLT is .90 (Van Den Burg & Kingma, 1999).

The primary stimulus was a 411‐word narrative about Pluto the dog, read aloud by participants (see Appendix A for narrative). To ensure consistency and standardized administration, participants read the narrative aloud from their laptop or computer screens, while standing up one step back from their desks. This allowed for gestures produced by participants (both hand and body gestures) to be accurately captured through the webcam and Zoom recordings. Instructions regarding the key encoding and recall tasks were also standardized (see Appendix A for the script of instructions for each gesture condition). In the Instructed Gesture condition, participants were asked to “show and tell using their hands” what was happening in the narrative as they read it aloud. Participants were not given any feedback on the types of gestures produced to maintain standardized administration and limit experimenter interference in gesture production. In the No Gesture condition, participants were asked to have their hands behind their backs. Finally, in the Spontaneous Gesture condition, participants were instructed to read the narrative aloud with no further instructions. Placing hands in pockets, or similarly holding their hands in a position that prevented hand movements, could inadvertently discourage spontaneous gesturing. The decision was made therefore to, if necessary, ask participants to remove their hands from that position, to best facilitate spontaneous gesturing without explicitly mentioning gesture. Consequently, where participants had their hands in their pockets, behind their backs, folded in front, or leaning on a chair or desk, they were asked to place their hands by their sides.

The study was initially designed to be conducted in a face‐to‐face setting. Due to the required physical distancing measures as a result of COVID‐19, however, the study was conducted online. Participants used the Zoom application on their laptop or computer with a working webcam and microphone, and this application was used to record both video and audio of the participants’ gestures and responses. Each participant was individually tested by the experimenter. Participants verbally consented to video and audio recordings prior to each portion of the recording being activated and were informed that these would be used for coding and scoring purposes (all participants consented for each portion). Participants were also required to take photos of their drawings of the RCFT and immediately email these to the researcher. All participants provided these images to the experimenter via email.

Participants first completed the RCFT copy trial. After completing the copy trial, participants were asked to scrunch up the paper after taking a photo of the drawing on their phones to (1) ensure that participants were not prompted by previous drawings in recall trials, and (2) send an email of the image to the experimenter immediately following the experiment for scoring purposes. Participants were then instructed to read aloud the narrative according to their allocated condition (Instructed Gesture, No Gesture, and Spontaneous Gesture). Participants read aloud the narrative within the 3‐min delay required between the RCFT copy and immediate trials. After reading the narrative aloud, participants completed the RCFT immediate trial. On completion of the RCFT Immediate trial, participants took a photo of their drawing on their phones and scrunched up the paper once again.

Following the RCFT immediate trial, the free recall question was asked (see Appendix B for the interview script). Participants were always asked the free recall question prior to the specific questions to avoid any prompting caused by the specific questions. After participants had answered the free recall questions, 16 specific questions were asked. Where participants got a question correct, they were asked the next open‐ended question. Where participants got an answer incorrect, were unsure of the answer, or gave an incomprehensible response, they were asked a follow‐up forced‐choice question. Participants were given nondirectional feedback, such as “thank you” following each response. All participants received specific questions in the same order to ensure standardization. Following the specific questions, the RAVLT learning trials were administered. After the learning trials, the interference trial was administered, followed by the immediate trial.

Following the immediate trial of the RAVLT, the RCFT delay trial was administered. Participants were again asked to take a photo of their drawings and then scrunch up the paper. Immediately following the RCFT delay trial, the RCFT recognition trial was administered, by the experimenter sharing their screen with the participant. Participants were then provided with a link to the online game Tetris (www.n‐blox.com), which was used as a filler task before the RAVLT delay trial. After 6 min of playing Tetris, participants were asked about their understanding of the purpose of the study. Although responses were not recorded, the majority of participants in all conditions stated they believed the study was about memory and the particular individual difference measures (RCFT and RAVLT), and no participants stated that they believed the study was about gestures. Once they had provided their answer, they were informed by the experimenter that the study aimed to assess whether producing gestures during the learning component of the narrative facilitated narrative recall.

The RAVLT delay trial was then administered, followed by the RAVLT recognition trial. Participants were thanked for their participation and awarded 60‐min course credit.

### 2.3 Transcribing, scoring, and coding

Transcribing, scoring, and coding of gestures and speech was done using Microsoft Word by the experimenter. All speech and gestures produced during the narrative learning and recall sections were transcribed verbatim prior to coding. This included any filler terms (e.g., “umm”). The total words spoken by participants during narrative learning was then calculated. Not all participants adhered perfectly to the scripted narrative, either skipping or adding words which lead to slight variability between participants in the total number of words spoken during encoding. The total number of words spoken was, therefore, used to calculate the rate of gestures produced per word spoken.

During recall, where participants’ responses demonstrated an accurate understanding of the narrative content, they were coded as correct. For example, if participants said Pluto was “excited” at the beginning of free recall, this was coded as correct for indicating that Pluto had been excited in the story. If participants said, “the turtle jumped on Pluto and into the water,” this was also coded as correct, as it indicated that participants had a conceptual understanding of the relevant event in the narrative, despite not using the precise correct wording. Where participants did not demonstrate either verbatim or conceptually correct recall or provided a nonresponse, this was coded as incorrect. For free recall, participants received 1 point for each part of the narrative they remembered correctly. Gestures were not coded for meaning during free recall, as it was not always clear which part of the story participants referred to in their gestures. Participants could receive a total of 66 points for correctly remembering the events and objects in the narrative (see Appendix A for free recall points breakdown).

Gestures produced by participants during the recall of specific questions were used to inform participants’ scores. That is, both speech and gesture were considered in determining whether answers were correct. Participants were given 2 points if they answered the open‐ended question correctly through speech and/or gesture, and 1 point if they answered the forced choice correctly through speech and/or gesture. Where both the open‐ended and forced‐choice questions were answered incorrectly, participants received a score of 0 for that item. Participants could achieve a total of 32 points for the specific questions (see Appendix B for specific questions).

The method used to transcribe and code gestures was based on the method by Dargue and Sweller (2018b, 2020), and the experimenter was trained prior to gesture coding. All gestures were first transcribed by the experimenter to accurately capture their form. Gestures were then categorized as iconic, beat, deictic, or metaphoric, according to McNeill's (1992) classification of gestures. This coding scheme has been used in previous papers by Alibali et al. (2001), Kartalkanat and Göksun (2020), and McKern et al. (2021). Where participants used deliberate body movements of the head, shoulders, or legs to demonstrate parts of the narrative (e.g., taking a step for “Pluto put his foot on the package” or looking down with the head for “Pluto looked down”), these were also recorded as gestures and categorized accordingly. Although theoretically, this may have occurred for all categories of gestures, in practice, this only occurred for iconic and deictic gestures. The above examples were coded as iconic and deictic gestures, respectively. As metaphoric gestures were not produced often by participants, leading to insufficient power to analyze them separately, iconic and metaphoric gestures were summed together to create the category of “representational gestures.” A similar classification of representational gestures has previously been implemented by Cameron and Xu (2011).

### 2.4 Reliability

A second coder independently coded 20% of recall transcripts and RCFT scores for inter‐rater reliability. Inter‐rater reliability was not needed for RAVLT scores, as scores were awarded where participants recalled the words correctly, with no subjective element to scoring. The coder was blind to each participant's condition for recall transcripts. A third coder independently coded 20% of encoding transcripts for gestures produced for inter‐rater reliability. Intra‐class correlations (ICCs) were used to assess reliability using an absolute agreement model. Only the first coder's scores were used in the final analyses, and thus the single measure ICC is reported. For Immediate RCFT recall, ICC = .983, *p* < .001, and for Delayed RCFT recall, ICC = .931, *p* < .001. For free recall, ICC = .955, *p* < .001, and for specific questions, ICC = .896, *p* < .001. For gestures produced during encoding, ICC = .996, *p* < .001 for representational gestures, ICC = .975, *p* < .001 for beat gestures, and ICC = .870, *p* = .005 for deictic gestures. Reliability for Immediate and Delayed RCFT, free recall, gestures produced during encoding, representational gestures, and beat gestures was high. Reliability for specific questions and deictic gestures was good.

### 2.5 Analysis plan

Stata was used for all analyses. A manipulation check using an independent samples *t*‐test was conducted, to assess whether participants in the Instructed Gesture conditions produced more gestures than participants in the Spontaneous Gesture condition. Results indicated that those in the Instructed Gesture condition produced significantly more gestures (*M* = 43.0, *SD* = 3.1) than those in the Spontaneous Gesture condition (*M* = .2, *SD* = .1), *t* = 14.02, *p* < .001, *η_p_* ^2^ = .77. To assess the effect of the two individual difference measures and gesture condition on recall measures, eight separate ANCOVAs with interactions were used for each individual difference and recall measure. Free recall scores and specific question scores were the dependent variables, and immediate and delayed verbal and nonverbal memory scores were the continuous predictors. Continuous predictors were centered on the mean prior to conducting analyses. Where follow‐up tests of simple effects were required, a Bonferroni adjustment was used to control the family‐wise error rate at alpha = .05. Finally, to evaluate the effect of gesture production at encoding on the recall of specific details of the narrative at test, a mixed‐effects ordinal logistic regression was conducted, due to the nested nature of the data. Individual gestures performed during encoding were matched to recall of those specific details at the test. That is, the unit of measurement for this analysis was gesture/recall point (fixed effect), nested within participant (random effect). The dependent variable was the score for each participant for each specific question.

## 3 Results

### 3.1 Preliminary analyses

Assumptions were checked and met for all ANCOVAs, with the exception of the interaction effects between (1) immediate verbal memory and gesture condition on free recall; (2) immediate nonverbal memory and gesture condition on free recall; and (3) delayed nonverbal memory and gesture condition on free recall. Residuals departed from normality for these three interaction effects. These three analyses were rerun with bootstrapping and the results were unchanged. For consistency of reporting, the original analyses without bootstrapping are reported. See Table 1 for the mean and standard deviation of the number of gestures produced by the gesture condition.

**Table 1:** Mean and standard deviation of number of gestures produced by gesture condition

Gesture condition	Mean	Standard deviation
Instructed Gesture	43.0	16.7
No Gesture	0	0
Spontaneous Gesture	.2	.7

As the distribution of gestures produced was skewed, Spearman's correlations were conducted to assess the relationship between gesture rate and verbal memory for each condition of gesture production. For those in the Instructed Gesture condition, the relationship between gesture production and immediate verbal memory was nonsignificant, *ρ* = .21, *p* = .264. The relationship between gesture production and delayed verbal memory was also nonsignificant, *ρ* = .21, *p* = .261. As the average number of gestures produced in the No Gesture condition was zero, Spearman's correlation produced no results. For those in the Spontaneous Gesture condition, there was also no significant relationship between gesture production and immediate or delayed verbal memory, *ρ* = –.30, *p* = .105, and *ρ* = –.35, *p* = .054, respectively.

### 3.2 The effect of gesture condition and individual differences on free recall

Four between‐subjects ANCOVAs with interactions were conducted. Each ANCOVA had an individual difference measure as a continuous predictor (immediate verbal memory [*M* = 11.74, *SD* = 2.50], delayed verbal memory [*M* = 11.66, *SD* = 2.66], immediate nonverbal memory [*M* = 19.57, *SD* = 19.47], and delayed nonverbal memory [*M* = 19.47, *SD* = 5.80]), with the total score on free recall as the dependent variable (Table 2).

**Table 2:** Mean and standard deviation of free recall and specific question score, by gesture condition

	Gesture condition					
Dependent variable	Instructed Gesture		No Gesture		Spontaneous Gesture	
	*M*	*SD*	*M*	*SD*	*M*	*SD*
Free recall	15.63	8.07	13.97	7.74	14.8	6.51
Specific questions	25.00	2.95	23.52	3.33	25.00	3.20

#### 3.2.1 Gesture condition and immediate verbal memory

There were no significant main effects of immediate verbal memory, *F*(1,84) = 2.12, *p* = .149, *η_p_* ^2^ = .025 or gesture condition on free recall, *F*(2,84) = .22, *p* = .805, *η_p_* ^2^ = .005. There was no significant interaction between gesture condition and immediate verbal memory on free recall, *F*(2,84) = 1.61, *p* = .206, *η_p_* ^2^ = .037.

#### 3.2.2 Gesture condition and delayed verbal memory

There were no significant main effects of delayed verbal memory, *F*(1,84) = 1.07, *p* = .304, *η_p_* ^2^ = .013 or gesture condition on free recall, *F*(2,84) = .26, *p* = .769, *η_p_* ^2^ = .006. There was no significant interaction between gesture condition and delayed verbal memory on free recall, *F*(2,84) = 1.94, *p* = .150, *η_p_* ^2^ = .044.

#### 3.2.3 Gesture condition and immediate nonverbal memory

There was a significant main effect of immediate nonverbal memory on free recall, *F*(1,84) = 4.73, *p* = .032, *η_p_* ^2^ = .053, such that higher levels of immediate nonverbal memory were associated with higher levels of free recall. There was no significant main effect of gesture condition, *F*(2,84) = .50, *p* = .611, *η_p_* ^2^ = .012 and no significant interaction between gesture condition and immediate nonverbal memory on free recall, *F*(2,84) = 1.69, *p* = .191, *η_p_* ^2^ = .039.

#### 3.2.4 Gesture condition and delayed nonverbal memory

There was no significant main effect of delayed nonverbal memory, *F*(1,84) = 3.94, *p* = .051, *η_p_* ^2^ = .045 or gesture condition on free recall, *F*(2,84) = .47, *p* = .626, *η_p_* ^2^ = .011. There was no significant interaction between gesture condition and delayed nonverbal memory on free recall, *F*(2,84) = 1.24, *p* = .296, *η_p_* ^2^ = .029.

### 3.3 The effect of gesture condition and individual differences on specific question recall

Four between‐subjects ANCOVAs with interactions were conducted. Each ANCOVA had an individual difference as a continuous predictor and recall of specific narrative points as the dependent variable (Table 2).

#### 3.3.1 Gesture condition and immediate verbal memory

**Fig. 1:**
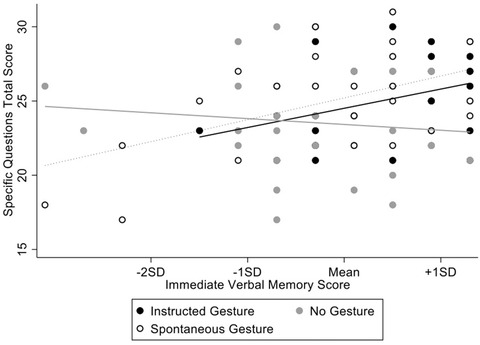
Interaction effect between gesture condition and immediate verbal memory on recall of specific narrative points.

There was a significant main effect of immediate verbal memory on recall of specific narrative points, *F*(1,81) = 5.16, *p* = .026, *η_p_* ^2^ = .060, such that higher levels of immediate verbal memory were associated with higher levels of recall. There was no significant effect of gesture condition on recall of specific narrative points, *F*(2,81) = 2.51, *p* = .087, *η_p_* ^2^ = .058. There was a significant interaction between gesture condition and immediate verbal memory, *F*(2,81) = 3.44, *p* = .037, *η_p_* ^2^ = .078 (Fig. 1).

Simple effects analyses comparing the Instructed Gesture, No Gesture, and Spontaneous Gesture conditions were conducted at one standard deviation below the mean, the mean, and at one standard deviation above the mean of immediate verbal memory. At one standard deviation below the mean, there were no significant differences between the Instructed Gesture and Spontaneous Gesture conditions, *F*(1,81) = .15, *p* = .697, *η_p_* ^2^ = .002, the No Gesture and Spontaneous Gesture conditions, *F*(1,81) = .005, *p* = .945, *η_p_* ^2^ = .000, or between the Instructed Gesture and No Gesture conditions, *F*(1,81) = .19, *p* = .658, *η_p_* ^2^ = .002. At the mean of immediate verbal memory, there were no significant differences between the Instructed Gesture and Spontaneous Gesture conditions, *F*(1,81) = .71, *p* = .404, *η_p_* ^2^ = .009, the No Gesture and Spontaneous Gesture conditions, *F*(1,81) = 4.97, *p* = .029, *η_p_* ^2^ = .058 (nonsignificant after Bonferroni adjustment), or between the Instructed Gesture and No Gesture conditions, *F*(1,81) = 1.66, *p* = .202, *η_p_* ^2^ = .020. At one standard deviation above the mean, there was no significant difference between the Instructed Gesture and Spontaneous Gesture conditions, *F*(1,81) = .67, *p* = .416, *η_p_* ^2^ = .008. There were significant differences, however, between both the Spontaneous Gesture condition and the No Gesture condition, *F*(1,81) = 9.36, *p* = .003, *η_p_* ^2^ = .104, as well as between the Instructed Gesture condition and the No Gesture condition, *F*(1,81) = 5.90, *p* = .017, *η_p_* ^2^ = .068, such that recall of specific points of the narrative was higher for those in both the Spontaneous Gesture and Instructed Gesture conditions than those in the No Gesture condition.

#### 3.3.2 Gesture condition and delayed verbal memory

**Fig. 2:**
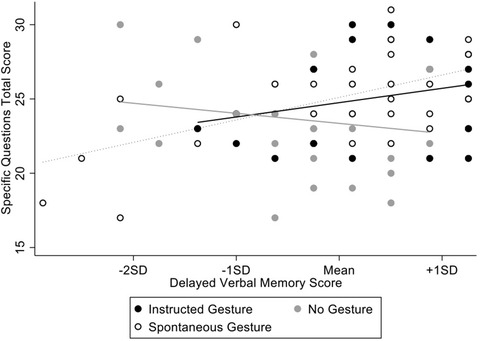
Interaction effect between gesture condition and delayed verbal memory on recall of specific narrative points.

There was no significant main effect of delayed verbal memory, *F*(1,81) = 3.24, *p* = .076, *η_p_* ^2^ = .038 or gesture condition on recall of specific narrative points, *F*(2,81) = 2.62, *p* = .079, *η_p_* ^2^ = .061. There was a significant interaction between gesture condition and delayed verbal memory, *F*(2,81) = 3.99, *p* = .022, *η_p_* ^2^ = .090 (Fig. 2).

Simple effect analyses comparing the Instructed Gesture, No Gesture, and Spontaneous Gesture conditions were conducted at one standard deviation below the mean, the mean, and one standard deviation above the mean of delayed verbal memory. At one standard deviation below the mean, there were no significant differences between the Instructed Gesture and Spontaneous Gesture conditions, *F*(1,81) = .03, *p* = .869, *η_p_* ^2^ < .001, the No Gesture and Spontaneous Gesture conditions, *F*(1,81) = .19, *p* = .662, *η_p_* ^2^ = .002, or between the Instructed Gesture and No Gesture conditions, *F*(1,81) = .04, *p* = .836, *η_p_* ^2^ = .001. At the mean of delayed verbal memory, there was no significant difference between the Instructed Gesture and Spontaneous Gesture conditions, *F*(1,81) = .18, *p* = .665, *η_p_* ^2^ = .002, the No Gesture and Spontaneous Gesture conditions, *F*(1,81) = 4.75, *p* = .032, *η_p_* ^2^ = .055 (nonsignificant after Bonferroni adjustment), or between the Instructed Gesture and No Gesture conditions, *F*(1,81) = 2.92, *p* = .091. *η_p_* ^2^ = .035. At one standard deviation above the mean of delayed verbal memory, there was no significant difference between the Instructed Gesture and Spontaneous Gesture conditions, *F*(1,81) = .74, *p* = .395, *η_p_* ^2^ = .009. There were, however, significant differences between both the Spontaneous Gesture condition and the No Gesture condition, *F*(1,81) = 10.56, *p* = .002, *η_p_* ^2^ = .115, as well as between the Instructed Gesture condition and the No Gesture condition, *F*(1,81) = 6.55, *p* = .012, *η_p_* ^2^ = .075, such that recall of specific points of the narrative was higher for those in both the Spontaneous and Instructed Gesture conditions than those in the No Gesture condition.

#### 3.3.3 Gesture condition and immediate nonverbal memory

There was a significant positive main effect of immediate nonverbal memory on recall of specific points of the narrative, *F*(1,81) = 5.88, *p* = .018, *η_p_* ^2^ = .068, such that higher levels of immediate nonverbal memory were associated with higher levels of recall. There was no significant main effect of gesture condition, *F*(2,81) = 1.38, *p* = .257, *η_p_* ^2^ = .033 and no significant interaction between gesture condition and immediate nonverbal memory on recall of specific points of the narrative, *F*(2,81) = .62, *p* = .539, *η_p_* ^2^ = .015.

#### 3.3.4 Gesture condition and delayed nonverbal memory

There was a significant positive main effect of delayed nonverbal memory on recall of specific points of the narrative, *F*(1,81) = 4.36, *p* = .040, *η_p_* ^2^ = .051, such that higher levels of delayed nonverbal memory were associated with higher levels of recall. There was no significant main effect of gesture condition, *F*(2,81) = 1.52, *p* = .225, *η_p_* ^2^ = .036 and no significant interaction between gesture condition and immediate nonverbal memory on recall of specific points of the narrative, *F*(2,81) = .93, *p* = .399, *η_p_* ^2^ = .022.

### 3.4 The effect of gesture production on recall of specific points of the narrative

A mixed‐effects ordinal logistic regression was conducted to assess the effect of gesture production at encoding on recall of specific narrative points. The three gesture conditions were merged for this analysis. The gesture production variables were dummy coded to indicate whether a representational, beat, or deictic gesture was performed at encoding while the participant read aloud the phrase in the narrative that corresponded to each specific question at recall (Table 3 ). The total number of gesture/specific question points is 1440, corresponding to 16 questions for each of the 90 participants (see Table 4 for frequencies by score).

**Table 3:** Frequencies and percentages of gestures performed at encoding points corresponding to specific recall questions, by gesture type

	Gesture type					
	Representational		Beat		Deictic	
Gesture performed	Frequency	Percentage	Frequency	Percentage	Frequency	Percentage
Yes	305	21.18	65	4.51	8	.56
No	1135	78.82	1375	95.49	1432	99.44

*Note*: Frequencies of “Yes” and “No” for gesture performed total to 1440 points, which is the total number of gesture/specific question points. Percentages are calculated accordingly.

**Table 4:** Frequency and percentage of scores achieved by participants across all recall questions

Specific question recall	Frequency	Percentage
0	130	9.05
1	411	28.60
2	896	62.35

*Note*: 0, 1, and 2 indicate the scores of 0, 1, or 2 achieved for each specific question. 2 indicates a correct answer to the initial open‐ended specific question, 1 indicates a correct answer to a follow‐up forced‐choice question, and 0 indicates an incorrect or no answer.

Of the potential 1440 points noted above, only 1437 are included in the analysis, as three points had missing recall data (see Method).

The overall model was significant, *Wald X* ^2^(3) = 14.82, *p* = .002. There was a significant positive effect of representational gestures on recall, *β* = .42, *SE* = .15, *z* = 2.72, *p* = .006, such that where a representational gesture was produced during encoding at that point in the narrative corresponding to that specific question, participants were more likely to have obtained a higher recall score than where a representational gesture was not produced during encoding. There was no significant effect of beat gestures on the recall of specific points of the narrative, *β* = –.07, *SE* = .26, *z* = –.27, *p* = .788. There was a significant negative effect of deictic gestures on recall of specific points of the narrative, *β* = –1.68, *SE* = .69, *z* = –2.43, *p* = .015, such that where a deictic gesture was produced during encoding at the point in the narrative corresponding to that specific question, participants were less likely to have obtained a higher recall score than where a deictic gesture was not produced during encoding.

Given the unexpected negative result for deictic gestures, the specific questions for which deictic gestures were performed were examined, to establish if this effect was due to unusual performance on one or a few individual recall questions. It was found that although the eight deictic gestures were spread across eight different questions, three of the gestures were performed by one participant. This participant performed one standard deviation below the mean for specific questions.

As verbal memory was found to be a significant moderator for the effect of gesture production on recall, two mixed‐effects ordinal logistic regressions with interactions between each gesture type and verbal memory were conducted post‐hoc to assess whether immediate and delayed verbal memory moderated the effect of the production of representational gestures, beat gestures, or deictic gestures on recall of specific points of the narrative.^1^

The overall interaction model, including immediate verbal memory, was significant, *Wald X* ^2^(7) = 21.22, *p* = .004. The pattern of main effects for the production of different types of gestures mirrored the initial analysis discussed above and are, therefore, not repeated here. There was a significant positive main effect of immediate verbal memory, *β* = .06, *SE* = .03, *z* = 2.15, *p* = .032, such that participants with higher immediate verbal memory were more likely to score higher on recall of specific points of the narrative. There was no significant interaction between gesture production and immediate verbal memory on representational, beat, or deictic gestures (see Table 5 for results of interactions between immediate and delayed verbal memory and production of different types of gestures on specific question recall).

**Table 5:** Interaction coefficient and significance of immediate and delayed verbal memory with gesture type on recall of specific points of the narrative

	Gesture type					
	Representational		Beat		Deictic	
Verbal memory	*β*	*p*	*β*	*p*	*β*	*p*
Immediate	–.00	.976	0.12	.289	.11	.729
Delayed	.00	.953	–.23	.020	.29	.417

**Fig. 3:**
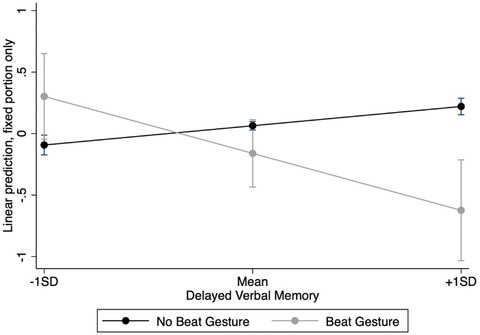
*Interaction effect between beat gesture production and delayed verbal memory on recall of specific narrative points*.

The overall interaction model, including delayed verbal memory, was significant, *Wald X* ^2^(7) = 25.15, *p* = .001. The pattern of main effects for the production of different types of gestures mirrored the initial analysis discussed above, and therefore, the results are not repeated here. There was a significant positive main effect of delayed verbal memory, *β* = .05, *SE* = .02, *z* = 2.00, *p* = .045, such that participants with higher delayed verbal memory were more likely to score higher on recall of specific points of the narrative. There were no significant interactions between representational gesture or deictic gesture production and delayed verbal memory on specific question recall (Table 5). There was a significant interaction between beat gesture production and delayed verbal memory, such that as delayed verbal memory scores increased, the effect of beat gesture production on the recall of specific points of the narrative decreased (Fig. 3).

Simple effects analyses were conducted comparing Beat Gesture production to No Beat Gesture production at one standard deviation below the mean, the mean, and one standard deviation above the mean of delayed verbal memory. There were no significant differences in specific recall for those who produced, and those who did not produce beat gestures either at one standard deviation below the mean, *β* = .40, *SE* = .34, *z* = 1.16, *p* = .248, or at the mean of delayed verbal memory, *β* = –.22, *SE* = .27, *z* = –.84, *p* = .400. The difference between those who did and did not produce beat gestures at one standard deviation above the mean of delayed verbal memory was nonsignificant after Bonferroni adjustment, *β* = –.84, *SE* = .41, *z* = –2.06, *p* = .039.

## 4 Discussion

The present study investigated the effect of gesture production at encoding on narrative recall, and whether this effect varied depending on levels of verbal and nonverbal memory. A further aim of the study was to determine whether there were differential effects of the production of different types of gesture on narrative recall, and whether gesturing during encoding supports the recall of the narrative as a whole or the recall of the specific points that the gestures are associated with. The results of this study suggest that gesture production at encoding may be beneficial for those with higher, but not lower, levels of verbal memory, albeit only at the recall of specific points of the narrative rather than at free recall. No significant results were found for nonverbal memory measures. The results also suggest that the production of representational gestures supports the recall of specific points with which gestures are associated.

### 4.1 Effects of gesture condition and individual differences on narrative recall

Contrary to expectations, no main effects of gesture condition, or interactions between gesture condition and measures of individual differences were found for free recall. Although this was consistent with the findings of Dargue and Sweller (2020) who found that gesture production during recall did not benefit free recall, it was inconsistent with previous findings where preschool children were found to benefit from gesture production (Cameron & Xu, 2011). There were, however, significant interactions between gesture condition and both immediate and delayed verbal (but not nonverbal) memory on recall of specific questions. This is inconsistent with past findings where gesture production during recall did not benefit the recall of specific points of a narrative (Dargue & Sweller, 2020). The difference in findings may have occurred as Dargue and Sweller (2020) assessed the effect of gesture production during recall only, whereas this study assessed the effect of gesture production during encoding. Effects of verbal memory are addressed here first, followed by nonverbal memory.

While there was no significant effect of immediate or delayed verbal memory on free recall, or significant main effect of delayed verbal memory on specific recall questions, as expected, there was a significant positive effect of immediate verbal memory on recall of specific points of the narrative. This significant main effect is not explored further here, however, considering the significant interaction between immediate verbal memory and gesture condition on recall of specific questions.

While an interaction was expected between verbal memory and gesture condition, it was anticipated that the beneficial effect of producing gestures during encoding would be greater for those with lower than higher levels of verbal memory (Goldin‐Meadow et al., 2001; Wagner et al., 2004). Unexpectedly, significant interactions were found in the opposite direction. While performance was higher in the Instructed and Spontaneous Gesture conditions than in the No Gesture condition, this effect was only for participants with higher, rather than lower, immediate and delayed verbal memory.

Consistent with expectations, as nonverbal memory increased, participants performed better on free recall. Findings in relation to recall of specific points of the narrative showed that those with higher immediate nonverbal memory performed better on recall of specific points of the narrative. This was also the case for those with higher delayed nonverbal memory. There was, however, no significant effect of delayed nonverbal memory on free recall (albeit at *p* = .051), suggesting that higher immediate nonverbal memory is associated with better performance on narrative recall than lower immediate nonverbal memory. Unexpectedly, however, there was no significant interaction effect between nonverbal memory and gesture on both measures of recall.

The finding that those with higher verbal memory performed better at recall when producing gestures suggests that gestures may be more beneficial for those with higher than lower verbal memory, and are inconsistent with the cognitive load hypothesis of gesture production, namely that gestures alleviate the load on working memory (Goldin‐Meadow et al., 2001). If gestures indeed lighten the load on working memory, it would be expected that an individual may be better able to encode and subsequently transfer newly learnt information into long‐term memory for later recall. This mechanism would, therefore, suggest that gestures should be more beneficial for individuals with lower verbal memory capacity. However, the current findings suggest that gestures were more beneficial for individuals with higher verbal memory capacity. As gestures and speech are part of an integrated system (McNeill, 1992), it may be that instructions prohibiting gesture in the No Gesture condition interfered with this system. In turn, those with higher verbal memory may have been unable to receive any additional benefit from gestures and were impaired by the inability to use gestures. This may have resulted in a similar performance on recall across the span of verbal memory capacity. As a result, those with higher verbal memory benefited more than those with lower verbal memory from the use of gestures in the Instructed Gesture condition and the Spontaneous Gesture condition, while performing similarly to those with lower verbal memory in the No Gesture condition on free recall.

The finding that gesture production did not differentially benefit those with different levels of nonverbal memory is inconsistent with findings by Wagner et al. (2004), who found that nonverbal memory was improved when gestures were used to explain a math problem while holding visuospatial items in participants’ memories. The current finding may indicate that while producing gestures can lead to mental simulations, as suggested by the GSA framework (Hostetter & Alibali, 2019), these mental simulations may not generate nonverbal memory traces. Therefore, the production of gesture and the benefit it has on the narrative recall may not be moderated by nonverbal memory. It may be that where gesture production is experimentally induced and does not occur naturally, it is more detrimental to learning than if gestures are spontaneously produced (Aldugom, Fenn, & Cook, 2020; Özer & Göksun, 2020b). Future studies should assess the differences in performance on verbal and nonverbal memory when gestures are manipulated and artificially produced, as compared with when they are produced naturally.

### 4.2 The effect of gestures on recall of specific points of the narrative

It was expected that participants would recall more for specific points of the narrative where they produced a gesture at encoding than for points where they did not produce a gesture at encoding. There was a significant positive effect of representational gestures on recall, such that participants were more likely to obtain a higher recall score for specific points at which they produced representational gestures during encoding than for points where participants did not produce representational gestures. There was no effect of beat gestures on recall. Unexpectedly, there was a negative effect of deictic gestures on recall, such that where deictic gestures were produced during encoding at the point in the narrative corresponding to a specific question, participants were less likely to score higher on recall than when deictic gestures were not produced during encoding.

Findings suggest that representational gesture production (metaphoric and iconic) during encoding is associated with better recall performance, consistent with findings that the production of representational gestures can be beneficial for learning and recall (Cameron & Xu, 2011; Cook et al., 2012). The current study expands on Cameron and Xu's (2011) findings to demonstrate that gesture production during encoding may be beneficial to recall. Results support previous gesture observation studies that found observing gestures benefits points in the narrative that the gestures accompanied, rather than recall as a whole (Dargue & Sweller, 2020), and suggest this effect can be extended to gesture production. In the present study, producing representational gestures may have reduced the cognitive burden of processing narrative content only through verbal working memory. This may have allowed participants to encode more of the narrative, consistent with the theory that gesture production lightens cognitive load (Goldin‐Meadow et al., 2001). Additionally, according to the GSA framework, gestures occur when mental simulations are generated during speech, and can allow speech content to be encoded imagistically (Hostetter & Alibali, 2019). In the current study, participants may have produced mental simulations while encoding the narrative, facilitating gesture production and allowing aspects of the narrative to be encoded through these mental simulations. However, participants were not explicitly instructed to produce gestures at specific points of the narrative. As a result, rather than facilitating recall of specific points at which gestures were produced, it may be that participants who produced gestures at those points were more likely to recall the specific points due to greater attention to detail. That is, a third variable may be present in the relationship between gesture production at encoding and recall of those specific points: greater attention to specific points during encoding may result in both increased likelihood of gesture production, as well as enhanced recall of those points. Future research should assess whether gesture production does in fact facilitate recall of specific points of the narrative by instructing participants to produce representational gestures at specific, predesignated, points.

The present findings also suggest that beat gestures do not necessarily support recall and learning, contrary to Vilà‐Giménez and Prieto's (2020) findings that beat gesture production during training was beneficial for 5‐ to 6‐year‐old children retelling narratives from an image. In Vilà‐Giménez and Prieto's (2020) study, participants first viewed videos of a narrator telling stories while using beat gestures, and were encouraged to pay attention to the narrator's hand movements. The improved storytelling at post‐test by children in the gesture‐encouraged condition may have been due to the benefits beat gestures may provide for narrative structure, rather than narrative recall. As the current study assessed narrative recall, rather than narrative structure, benefits from beat gesture production may not have been evident. Current findings reflect the heterogeneity of previous results highlighted in the systematic review conducted by Vilà‐Giménez and Prieto (2021), who found that in only six of 11 studies, beat gestures were beneficial to narrative development. It is important to note that some studies assessed the impact of beat gesture observation on later narrative performance, and secondly, the studies assessed the use of beat gestures at both focal content words (e.g., an object or action) and discourse markers (e.g., therefore, because, but). In the present study, participants were told to use their hands, but were not specifically instructed on the types of gestures to produce when reading the narrative.

The finding that deictic gesture use results in lower performance in the present study appears at first glance to be inconsistent with Cameron and Xu's (2011) finding, where such gestures were found to benefit memory recall. Current findings may have differed from Cameron and Xu (2011), as they assessed deictic gesture production during recall, whereas the current study only assessed gesture production at encoding. Furthermore, although participants in the Instructed Gesture condition produced more deictic gestures as a whole, very few of these deictic gestures corresponded to the points of the narrative which were assessed through specific questions. Therefore, no firm conclusions can be drawn from the findings, as only eight deictic gestures were produced during encoding in relation to points which were subsequently queried through the specific questions. Future research should assess the impact of deictic gesture production during encoding on subsequent recall, to determine whether there is a beneficial effect of deictic gestures on recall.

An exploratory analysis assessing the interaction between verbal memory and types of gestures produced on recall of specific points in the narrative was conducted. Findings indicated that there was no interaction between representational or deictic gestures and verbal memory on recall. There was a significant interaction between delayed verbal memory and beat gesture production, such that as participants’ levels of delayed verbal memory increased, those who produced beat gestures were less likely to obtain higher scores on specific recall. This finding indicates that where individuals have better verbal memory resources, the production of beat gestures may be detrimental to recall. This finding supports previous findings where participants with lower levels of verbal memory performed worse at recalling the names of objects from pictures when they were prevented from gesturing, and nonmeaningful (or beat) gestures did not appear to support word retrieval (Pyers, Magid, Gollan, & Emmorey, 2021). The finding in the current study that participants who produced representational gestures were more likely to recall specific points of the narrative also supports Pyers et al. (2021) findings, where producing more representational gestures was associated with a higher recall of object names. However, it is important to note that while in the current study participants produced gestures during the encoding of a narrative, in Pyers et al. (2021) study, participants’ gesturing was assessed during the retrieval of an object name from a picture.

### 4.3 Limitations and future directions

As the present study did not systematically vary the types of gestures produced by participants, it is unclear whether the types of gestures may differentially benefit recall (i.e., whether iconic gestures may benefit recall more than beat gestures). Future research should explore the differential benefits of different types of gestures, such as the difference between beat and iconic gestures, by systematically varying the type of gestures participants are required to produce during encoding. The present study did not assess the relevance of the gestures to the corresponding speech due to very low gesture rates in the No Gesture and Spontaneous Gesture conditions, potentially missing any effects of semantic relatedness or temporal synchrony. Future research may benefit from having a naïve coder assess the relevance of gestures and temporal synchrony of gestures to corresponding speech. As the current study only assessed gesture production at encoding, it is unclear whether certain types of gestures may be more beneficial to learning when produced at different times of the learning process. Future studies should assess the differential impacts of gesture production during encoding and recall, to determine when gesture production may best support learning.

As the present study did not measure cognitive load during gesture production, it cannot be determined whether the cognitive load was differentially lightened for participants with different levels of verbal and nonverbal memory. Future studies should aim to assess whether the cognitive load is differentially lightened for those with different levels of verbal or nonverbal memory, to more accurately investigate the benefits of gesture production. For example, a secondary task could be used (e.g., Wagner et al., 2004), which would add a cognitive burden on top of the primary task of interest. The effects of gesture production could be examined with secondary tasks of varying difficulty, such as remembering word lists of different lengths. Finally, given the theory that gesture lowers the load on working memory specifically, future studies should explore whether the effects of gesture are moderated by working memory (rather than general memory) measures specifically.

### 4.4 Conclusion

Through highlighting that gesture production during encoding can benefit narrative recall, particularly for individuals with high verbal memory, the current study extends our understanding of whether and when gesture production enhances narrative recall. Additionally, the production of representational gestures during encoding facilitated recall of the narrative for the specific points which they accompanied, whereas beat gestures had no effect, and deictic gestures were shown to have a detrimental effect on recall. Such differential effects highlight that the production of certain gestures may be more beneficial to recall than others: gestures may lighten cognitive load most when they are semantically related to the content of accompanying speech.

## Note

1 The authors thank an anonymous reviewer for suggesting this exploratory analysis.

## Appendix A Pluto Narrative Text, Instructions, and Points

### A.1

Instructions: For the next task, I would like to record both audio and video. Please stand one step back from your desk.

**Instructed gesture**: Please read this narrative out loud. I would also like you to show and tell using your hands, as you read it aloud.

**No gesture**: Please read this narrative out loud. I would also like you to keep your hands behind your back as you read it aloud.

**Spontaneous gesture**: Please read this narrative out loud.

Pluto's Surprise Package

**Pluto** the **dog** was **excited** to receive some **mail**. Pluto **ran outside** to open his **letterbox** and found a big **package** inside. Pluto put the package in **his mouth** and placed it **on the ground**. Then, the package **started to move**. All of a sudden, **four legs** popped **out of the package**.

While Pluto made a **pile of his other letters** on the ground, the package started to **walk away**. Pluto **stretched out his** leg and **stepped on the package** to try and stop it from **moving**, but a **gust of wind** picked his letters up and **blew them all around**. Pluto was **strugglin**g to **catch all of his letters** and **keep the package still**.

The package started **dragging Pluto away** from his letters. Pluto then **picked up** a **big rock** in his mouth and **put the rock on the package** to **stop it from walking** any further. Suddenly, **Pluto's letters flew past him** in the air, and he **chased** them, **barking** loudly. While Pluto was chasing his letters, the **package opened,** revealing a **turtle** inside. The turtle **walked out of the box**, but Pluto **did not see the turtle** and **tripped over it** while **chasing his letters**.

Pluto tumbled over the turtle **toward the edge of a cliff**. Pluto **stopped** just in time, and **managed to catch all of his letters** in his mouth **before they fell off the ledge**. Pluto **looked down**, and saw a **lake full of water**. This **scared** Pluto. Pluto held onto **the edge with his legs** as best as he could, **but the turtle walked on Pluto's back** and **dove off** into **the water below**, causing Pluto to **fall into the lake** with the turtle **and his letters**. Pluto was **very angry** at the turtle **for making him fall into the water and lose his letters again**.

The **turtle swam around Pluto in circles [happily]**, and then **climbed out** of the water **onto a beach**. Pluto **chased the turtle out** of the water, barking angrily. The turtle then **reached into his shell**, and **got out all of Pluto's letters**. The turtle then **dried off** Pluto's letters. This **made Pluto very happy**. The turtle **then started walking**. Pluto **followed** the turtle, and **picked up both the turtle and his letters in his mouth**. They **smiled** at each other, and **walked back to Pluto's house** together happily as **friends**.

*Bold = free recall point (*N* = 66).

## Appendix B Interview Script with Free Recall and Specific Questions

### B.1

Interview Questions

Now, I am going to ask you some questions about the story you read earlier. If you do not know the answers you can just guess, ok?

**Free Recall Question**: First, tell me everything you remember about the story you read earlier.

Now, I am going to ask you a few more questions about the story you just read. The questions I am going to ask will not necessarily be in the same order as what you read in the narrative.

1. Where did Pluto run to at the start of the story?
a. Did Pluto run to his mailbox or his house?
2. How did Pluto feel when he received some mail?
a. Did Pluto feel sad or excited?
3. What made Pluto tumble over?
a. Did the turtle or a rock make Pluto tumble over?
4. What started dragging Pluto away from his letters?
a. Did the wind or the package drag Pluto away from his letters?
5. How did Pluto feel when he got his letters back?
a. Did Pluto feel happy or surprised when he got his letter back?
6. Where did Pluto and the turtle walk back to in the end?
a. Did they walk back to a park or Pluto's house?
7. What did Pluto find in his letterbox?
a. Did Pluto find a package or flowers in his letterbox?
8. What popped out of Pluto's package?
a. Did balloons or legs pop out of Pluto's package?
9. What did the package start to do when Pluto was making a pile with his letters?
a. Did the package start walking away or did it break open?
10. What did Pluto put on the package to try and stop the package moving?
a. Did Pluto put his letters on the package or did he put a rock on the package to try and stop it from moving?
11. What did the wind do to Pluto's letters?
a. Did the wind blow the letters all around or did the wind blow the letters into a pile?
12. What animal was inside Pluto's package?
a. Was there a kitten or a turtle inside the package?
13. What could Pluto see at the bottom of the cliff?
a. Did Pluto see water or trees at the bottom of the cliff?
14. What happened to Pluto when he was on the ledge of the cliff?
a. Did Pluto manage to stop himself from falling off the cliff or did Pluto fall into the water?
15. What was the turtle doing in the water?
a. Was the turtle swimming in circles around Pluto or was the turtle splashing Pluto with water?
16. What did the turtle do to Pluto's wet letters?
a. Did the turtle rip the letters up or did the turtle dry off Pluto's letters?
